# Psychometric properties of factors influencing Healthcare Career Choice Scale

**DOI:** 10.1002/nop2.541

**Published:** 2020-06-14

**Authors:** Hayfa Almutary, Modi Al‐Moteri

**Affiliations:** ^1^ Medical/Surgical Department Faculty of Nursing King Abdulaziz University Jeddah Saudi Arabia; ^2^ Nursing Department Faculty of Applied Medical Sciences Taif University Taif Saudi Arabia

**Keywords:** career choice, career decision‐making, nurses, nursing, workforce

## Abstract

**Aim:**

The aim of this paper was to modify Factors Influencing Teaching Choice scale (FIT‐Choice scale) to be appropriate for healthcare professionals and to assess the psychometric properties of the modified scale.

**Design:**

A cross‐sectional study was used.

**Methods:**

A convenience sample of 395 students at the preparatory stage of their health path participated in study to evaluate the psychometric properties of “Healthcare Career Choice Scale” (HCC Scale). The study involves two phases: instrument modification and psychometric evaluation.

**Results:**

The content validity index was 0.88. Using factor analysis, 12 factors were extracted and explained 71.8% of the total variance. The internal consistency was demonstrated with Cronbach's α = .91.

## 1 INTRODUCTION

Career decision‐making is one of the most complex and critical activities that undergraduate first‐year students will make. Indeed, this most important decision will shape their future. The students identify their future career through a process of estimating their abilities, skills and work values (Brown, 2002).

Factors influencing a healthcare professional's career choice have been investigated worldwide from different perspectives such as (a) students’ perception (Al‐Humaidan & Mersal, 2017; Liaw, Wu, Chow, Lim, & Tan, 2017); (b) gender difference (Kawamoto et al., 2016; Liaw et al., 2017); and (c) family support (Neilson & McNally, 2013; Puertas, Arósquipa, & Gutiérrez, 2013). These studies are motivated by the growing literature indicating that there is critical shortage in the health workforce across and in most countries (Goel, Angeli, Dhirar, Singla, & Ruwaard, 2018; Wu, Low, Tan, López, & Liaw, 2015) and there are disparities in the health workforce in the health labour market. Globally, nursing shortages are considered problematic worldwide (Liaw et al., 2017). Further, the problem of health workforce shortages differs from one specialty to another. University policymakers need to explore the comparatively attractive factors influencing career decision‐making of prospective healthcare professionals, so that they can consider these factors during student selection and set appropriate career counselling programmes.

## 2 OVERVIEW OF EXPECTANCY‐VALUE MODELS

Many theories and models have been developed to understand career choice. One of these models is the expectancy‐value model of career choice which has been found to be comprehensive and based on empirical evidence (Watt & Richardson, 2007). The first version of expectancy‐value motivation models was proposed by Atkinson in 1958. The underlying principle of this model was to understand “how beliefs are combined” to form behaviour. Three decades later, the model was revised by Eccles et al. (1983) to better understand students’ career choices and their academic performance. In the revised model of expectancy‐value, students’ career decisions can be assessed by investigating their perceptions and values. Generally, three constructs make students place a higher value on a selected career: intrinsic value, utility value and attainment value (Eccles et al., 1983).

The expectancy‐value model of career decision‐making has been extensively used in other fields such as science, technology, engineering and mathematics to predict students’ selections (Wang & Degol, 2013). Perhaps the most popular adaptation of the expectancy‐value model (Eccles et al., 1983) is Watt and Richardson’s (2007) theoretical model. Their model captures the perceived individual abilities required for success and management of potential challenges. In the Watt and Richardson’s (2007) model, value component includes the perceived intrinsic value (interest in selecting a particular profession), personal utility value (e.g. job security) and social utility value (e.g. desire to help others and make a social contribution). In addition to value components, Watt and Richardson (2007) model includes constructs to evaluate individuals’ perception of their abilities in the career of interest. Indeed, social status, prior experience, learning experience and persuasion by others are all considered as factors that may shape career selection (Watt & Richardson, 2007).

Watt and Richardson (2007) designed a scale to measure factors influencing the selection of teaching as a career. This scale called Factors Influencing Teaching Choice Scale (FIT‐Choice scale) and consisted of 58 items with 18 factors (Watt & Richardson, 2007). These include the following: five factors concerning perception of the profession with 17 items (social status, difficulty, expertise, social dissuasion, salary), one factor for career choice satisfaction with two items and twelve factors concerning motivation “(ability, intrinsic career value, fallback career, job security, time for family, job transferability, shape future of children/adolescents, enhance social equity, make social contribution, work with children/adolescents, prior teaching and learning experiences and social influences)” (Watt & Richardson, 2007: p. 175). The possible responses to the FIT‐Choice scale are based on a 7‐point Likert scale. The scale has been validated previously in many studies (Eren & Tezel, 2010; Watt & Richardson, 2007, 2008).

The FIT‐Choice scale is characterized by its “ability to synthesize multiple theoretical perspectives, capture the key components of what motivates an individual and explain a wide range of achievement‐related behaviors” as well as barriers preventing the individual from engaging in the task (Barron & Hulleman, 2015: p. 2). It seems that adaptation of FIT‐Choice scale is useful in the health context as the basis of this theoretical model is appropriate to understand motivation related to healthcare professionals’ career selection. Hence, the aim of this paper is to modify FIT‐Choice scale to be appropriate for healthcare professionals and to assess the psychometric properties of the modified scale.

## 3 METHODS

The psychometric property testing involved two phases: modification of the FIT‐Choice scale and evaluation of the psychometric properties of the newly modified instrument.

### 3.1 Phase 1: Instrument modification

An approval was granted from the primary author to use the FIT‐Choice scale for healthcare professionals. FIT‐Choice scale was specifically designed to serve a teaching population. It is therefore important to modify the scale items to be suitable to assess factors influencing prospective healthcare professionals’ career decision‐making. To modify the scale, eight subject matter experts (SMEs) were employed on the basis of their area of expertise and qualifications to review the FIT‐Choice scale and make the required modification. Three steps were applied to gain an instrument that is appropriate to understand motivation related to healthcare professionals’ career selection.

#### 3.1.1 First step: Rewording

The SMEs were asked to ensure that the scale items and framework were relevant in addition to reviewing the wording of the items. The modification includes the following: replacing “teacher and words relating to teaching” by words related to health professionals. For example, the phrase “I have the qualities of a good teacher" was replaced by "I have the qualities of a good nurse,” “I have the qualities of a good physiotherapist”, etc. (see Appendix S1).

#### 3.1.2 Second step: Assessing content validity

To measure content validity, content validity index (CVI) and content validity ratio (CVR) were both calculated. The CVI was calculated to assess the relevancy and the clarity of each item (I‐CVI) and the overall scale (S‐CVI) (Shrotryia & Dhanda, 2019). For CVI, the eight experts were asked to independently answer the following questions: to what extent is this item relevant to understand motivation related to healthcare professionals’ career selection? A four‐point scale was used to avoid a neutral point: 1 = irrelevant, 2 = slightly relevant, 3 = moderately relevant and 4 = highly relevant. The item‐level (I‐CVI) was calculated by summing the number of experts who scored the item as 3 or 4 and then divided by the total number of experts (Polit & Yang, 2016). The I‐CVI for all the items of the scale ranged from 0.64–1. An I‐CVI <0.78 was excluded (Polit & Beck, 2006). Of the 58 items of original FIT‐Choice scale, 55 items scored above 0.78 and were founded to be relevant to understand motivation related to healthcare professionals’ career selection. Three items (Item Nos: 8, 14 and 16) scored below 0.78 and were excluded, as these items were considered irrelevant. The S‐CVI was then calculated to ensure content validity of the overall scale. According to Polit and Beck (2006), minimum S‐CVI should not be <0.8 to ensure content validity. The S‐CVI was 0.88 indicating high content validity.

To specify whether an item is necessary for understanding motivation related to healthcare professionals’ career selection, CVR was calculated (Shrotryia & Dhanda, 2019). The eight experts were asked to give a rating from 1–4 to each item ranging from 1 = definitely essential, 2 = maybe essential, 3 = useful, but not essential and 4 = not essential. Six items of 58 items (Item Nos: 2, 4, 20, 24, 34 and 38) were rated as “not essential” by five of the experts as their CVR was negative and were excluded. For the remaining items, the rating ranged from 0–1 and was considered essential. Consequently, the modified version included 49 items (see Supplementary file I).

#### 3.1.3 Third step: assessing face validity and scoring response

Following instrument modification, a cognitive testing of the statements was conducted on a purposively selected sample of five students (Willis, 1994). The test is a form of structured concurrent think‐aloud interviewing used to uncover the cognitive processes that occur as respondents think about their answers to scale questions. This test was used specifically to clarify comprehensibility and response format. A few issues concerning the clarity of the instructions were identified during the cognitive interviewing technique and consequently were amended. Also, a change was introduced into the scoring system: instead of using a 7‐point Likert scale, a 5‐point Likert scale, which ranged from 5 (extremely important)–1 (not at all important), was used. This change was aimed to reduce participants’ “annoyance level” and to increase response rate. The participants also indicated that the time needed to complete the instrument was approximately 20 min. The new modified instrument was named as “Healthcare Career Choice Scale” (HCC Scale) to reflect the career choice of the targeted population.

### 3.2 Phase 2: psychometric evaluation

#### 3.2.1 Study design and participants

A cross‐sectional study was used where an online survey was conducted to evaluate the psychometric properties of the HCC Scale. The survey was made available online in the College of Applied Medical Sciences webpage from February 2018–April 2018 for participants who enrolled in the health studies track and were in their first year of their study. Participants were eligible for this study if they were as follows: (a) in the first year at the university (preparatory stage); (b) enrolled in a health path (i.e. nursing, medical laboratory, physiotherapy and radiology); and (c) willing to participate in this study. Students who enrolled in the other non‐medical paths at the university were excluded. In total, 500 male and female students were invited to participate in the survey. Non‐probability sampling technique was used. At least 300 participants were considered necessary to detect a value of an intra‐class correlation efficient (ICC) (Tabachnick & Fidell, 2014). The rule of 1:5 was also considered, which is recommended to calculate sample size for scale development. Given this, 58 items of the instrument require at least 290 participants. Recruitment was ceased when the number of participants exceeded 300. Of the 500 eligible participants, 395 agreed to participate and complete the online survey.

#### 3.2.2 Instruments

The Healthcare Career Choice Scale (HCC Scale) was used in data collection to evaluate the psychometric properties of the scale. In addition to the 49 items, the scale included data on demographics such as age, gender, health specialty, General Aptitude Test (GAT) and Standard Achievement Admission Tests (SADT).

#### 3.2.3 Ethical consideration

Research Ethics committee approval was granted for this study from the research committee at the university (No: 40‐34‐0060). Participant identifiers or personal information was not collected as part of the study. Data were collected as anonymous individuals, and study data were transferred and stored in a secured place.

#### 3.2.4 Data collection

After obtaining the research Ethics committee approval, a link for the self‐reported online questionnaire was distributed. Logging onto the link opens explanatory statements followed by participation consent with two option icons (Proceed and Exit). Only students who were interested in the study completed the online survey.

#### 3.2.5 Statistical analyses

Data were analysed using IBM SPSS Statistics version 22 (IBM Corporation). Demographic information is reported descriptively as number (*N*), percentage (%), mean and standard deviation (*SD*) as appropriate. In addition, the construct validity of the HCC Scale was evaluated using exploratory factor analysis (EFA) with the principal component analysis and promax rotation method. This method of rotation was chosen since the items of the HCC Scale were assumed to be correlated with each other. Three criteria were used to determine the number of factors to be retained: “(a) having eigenvalues above 1; (b) the scree plot; and (c) interpreting both pattern and structure of each factor.” Items were retained when factor loadings were ≥0.45, were not cross‐factor loaded and conceptually fit with the individual factor (Polit & Yang, 2016). Finally, the internal consistency of the total scale and these factors was measured and reported using Cronbach's α.

## 4 RESULTS

### 4.1 Participants’ characteristics

A total of 395 participants responded to the surveys, giving a response rate of 79%. Participants’ characteristics are summarized in Table 1. The mean age of the participants was 19.07 (*SD *= 62), ranging from 18–21 years. More than half of the participants were female (58.7%). The mean results of General Aptitude and Standard Achievement Admission Tests were similar (m = 79). More than quarter of the participants were enrolled in nursing (28.4%).

**TABLE 1:** Participant characteristics (*N* = 395)

Characteristics	Mean (*SD*)	Number (*N* = 395)	Percentage, %
Age (years)	19.07 (0.62)		
GAT	79.18 (4.29)		
Standard Achievement Admission Test	79.92 (5.05)		
Gender, *N* (%)			
Male		163	41.3
Female		232	58.7
What programme are you enrolled in?			
Nursing		112	28.4
Laboratory		80	20.3
Radiology		100	25.3
Physiotherapy		103	26.1

Abbreviations: GAT, General Aptitude Test; *SD*, standard deviation.

### 4.2 Construct validity

Few missing data were found in the overall scale (˂5%). The pattern of missing was evaluated by Little's MCAR test (chi‐square = 3,447.08, *df* = 3,502, *p* = .743). The results indicated that data were missing completely at random. Consequently, the expectation maximization imputation was applied to substitute missing data. Using this method yields unbiased estimates if there are little missing data (Musil, Warner, Yobas, & Jones, 2002).

The results revealed that the Kaiser‐Meyer‐Olkin (KMO) test of sampling adequacy was 0.88 and the Bartlett's test of sphericity was 10,535.2, *df* = 1,035, *p* < .001, which was appropriate for factor analysis and indicted the existence of common factors between the variables.

Using factor analysis, 12‐factor was extracted, and these explained 71.8% of the total variance. The factor loadings ranged from 0.47−0.93, demonstrating the actual correlation between each item and the factor scores. In addition, the result was confirmed through the scree plot, which revealed marked discontinuity after the 12th factor. Six items were removed (Item Nos: 9, 11, 19, 28, 34 and 44) because they were not loaded on the pattern coefficients. But examining the structure coefficients showed that these items either had cross loaded on multiple factors and/or had loading values <0.45. Consequently, a total of 43 items loaded into 12 factors were retained. These factors were named “contribution to society,” “social status,” “career value and perceived abilities,” “work with patients,” “satisfaction with choice,” “job security,” “prior experiences,” “qualities,” “social influences,” “fallback career,” “difficulty” and “social dissuasion.”

The number of items in each factor ranged between 2–5 items. Cronbach's alpha of the 43‐item of HCC Choice Scale was 0.91 and the 12 factors ranged from 0.64–0.91 (Table 2). The first factor “make social contribution” accounted for 26.78% of the variance.

**TABLE 2:** Factor analysis of the 43 items, and Cronbach's a subscale reliabilities

Items	Factor loading												Cronbach alpha (α)
	1	2	3	4	5	6	7	8	9	10	11	12	
Factor 1: Contribution to society													
This health specialty allows me to offer service to society	0.81												.89
This health specialty makes a worthwhile social health contribution	0.80												
This health specialty will allow me to benefit the socially needy people	0.70												
This health specialty enables me to ‘give back’ to society	0.65												
This health specialty allows me to have an impact on people health	0.49												
Factor 2: Social status													
I think people working in this health specialty feel appreciated by society		0.82											.82
I think this health specialty is a well‐respected career		0.71											
I think people working in this health specialty feel their occupation has high social rank		0.69											
I believe this health specialty is perceived as professionals		0.64											
I believe this health specialty is perceived as a high‐status occupation		0.62											
Factor 3: Career value and perceived abilities													
This health specialty suited my abilities			0.91										.87
I have good skills specific for this health specialty			0.86										
I have the qualities needed to succeed in this health specialty			0.75										
I am interested in this health specialty			0.70										
I’ve always wanted to select this health specialty			0.50										
Factor 4: Work with patients													
I want to work in patient‐centered environment				0.93									.91
I like working with patient of all ages				0.90									
I want a job that involves working with patients of all ages				0.82									
Factor 5: Satisfaction with choice													
I’m satisfied with my selection of my current health specialty					0.92								.91
I carefully thought before selecting my current health specialty					0.90								
I’m happy with my decision about selecting this current health specialty					0.80								
Factor 6: Job security													
This health specialty provides a reliable income						0.88							.82
This health specialty will offer a steady career path						0.79							
This health specialty will provide me a secure job						0.67							
This health specialty is well paid						0.55							
People working in this health specialty earns a good salary						0.54							
Factor 7: Prior experiences													
In this health specialty, I have seen some good role‐models							0.89						.89
I have had positive health care experience							0.82						
I have been amazed by some inspirational people at this health specialty							0.77						
Factor 8: Qualities													
I think this health specialty requires high levels of expert knowledge								0.90					.79
I think this health specialty need high levels of procedural knowledge								0.81					
I think this health specialty requires high level of specialized knowledge								0.73					
Factor 9: Social influences													
My family think I should study this health specialty									0.92				.80
People I’ve met think I should study this health specialty									0.87				
My friends encourage me to study this health specialty									0.67				
Factor 10: Fallback career													
I chose this health specialty as a last‐resort career										0.73			.64
I was not accepted into my first‐choice career										0.72			
I was unsure of what health specialty I wanted										0.71			
Factor 11: Difficulty													
I think this health specialty is hard											0.89		.70
I think people working in this health specialty have heavy workload											0.81		
Factor 12: Social dissuasion													
I was encouraged to select other than this health specialty												0.91	
I was told by others that selecting this health specialty was not a good decision												0.63	
I was influenced to consider other career than this health specialty												0.47	
Total													.91

## 5 DISCUSSION

Shortage of well‐trained healthcare professionals has been acknowledged worldwide, specifically in nursing professions. Because nurses represent a large portion of healthcare system, the shortage of nurses has adverse effects on a healthcare system. However, misconceptions about the nature of nurses’ work and the lack of social recognition discourage students from selecting nursing as a profession (Liaw et al., 2016). The competition among healthcare programmes and schools to attract first‐year college health students prompts attention to the measurement of healthcare specialties preferences among undergraduate healthcare students. This perhaps provides policymakers with important information for developing policies to increase the number of healthcare professionals, in particular in nursing enrolment. Hence, this study aimed at examining the psychometric properties of the HCC Scale to establish one instrument able to address the deficiencies of the currently available scales in the health literature.

As described above, the scale was developed based on the FIT‐Choice scale (Watt & Richardson, 2007), which has a solid framework that can capture different domains relevant to the students’ preference about career choice. Screening students’ career choice early during their enrolment has many benefits. It may help decision‐makers understand factors that have an impact on student career decision. This in turn provides necessary understanding required to manage the disparities in the health workforce in the labour market. In addition, using this instrument may enable prediction of future trends in healthcare systems globally.

The results of this study provide initial evidence about the validity and reliability of the HCC Scale which can be used to assess healthcare specialties preferences among undergraduate students. The HCC Scale was initially reviewed by 8 experts, who agreed that the 49 items were relevant and were capable of measuring students' preferences for their prospective healthcare career choice. The content validity was also assessed through asking those experts to rate the instrument items. The results show that the S‐CVI was 0.88 indicating the validity of the instrument. Additionally, the face validity was established through a pilot test using a structured concurrent think‐aloud interviewing technique to uncover the cognitive processes that occur as respondents think about their answers to scale questions. Evidence supports using this technique in validation studies to discover any potential problems (Alshammari, Alhadreti, & Mayhew, 2015; Padilla & Leighton, 2017). Therefore, using this technique has refined our modified scale to clarify comprehensibility and response format.

The construct validity of the HCC Scale was further demonstrated using factor analysis. The items were loaded into 12 different factors that had meaningful constructs. Instrument modifications were made based on factor analysis to eliminate items with weak loading or that were unrelated. A high factor loading of ≥0.45 was used to determine the included item in each factor to gain a more robust instrument and discriminate among items. Accordingly, the HCC Scale included 43 items loaded into 12 factors. Each factor was named to reflect the content of each construct, and these were “make social contribution,” “social status,” “intrinsic career value and ability,” “work with patients,” “satisfaction with choice,” “job security,” “prior experiences,” “expertise,” “social influences,” “fallback career,” “difficulty” and “social dissuasion.”

From the 12 motivational factors, the two social utilities related values were rated highly: “work with patients” was the highest rated followed “by make social contribution.” This was not surprising, since community*‐*based healthcare services are integral parts of the health care* *delivery system (Foot et al., 2014). This result is also similar to previous report where the work culture was the most influential factor of specialty choice among medical students (Chang, Hung, Wang, Huang, & Chang, 2006). Previous studies also recognized professional security as one of the influential factors of career selection among students (Al Subait et al., 2017; Liaw et al., 2016). In a qualitative study that investigated healthcare students’ choice of nursing as a career, it was found that personal preference, prior experience and job security were the main emerging themes that influence career choice (Liaw et al., 2016). “Fallback career” was rated low indicating that participants were enrolled in their first choice and had not chosen their career because they were unable to enrol in another option.

### 5.1 Strengths and limitations

The main strength of this study was the development of a comprehensive instrument that is driven from an expectancy‐value model of career decision‐making. Another strength was using a large sample size sufficient to conduct the factor analysis. As in any study, some limitations were identified and are here acknowledged. The sample of this study was recruited from one public university, and ideally there should be involvement of other university populations in the study to increase generalizability of the results. Future studies need to replicate this study and assess other validity features such as the convergent and divergent validity.

### 5.2 Relevance to nursing practice, education or research

Understanding students’ career choice by screening early during their enrolment can potentially help decision‐makers understand factors that may influence career choices and assist in managing the disparities in the health workforce in health labour market. Using this instrument may help the schools of nursing to identify and attract prospective nursing students to join and continue the programme thereby increasing the number of nurses in the workforce. In addition, using this instrument may enable predictions of future trend in healthcare careers globally.

## 6 CONCLUSION

The underlying theoretical background of the scale is robust for identifying factors that influence achievement‐related behavioural choices. The psychometric properties of the HCC Scale have been assessed which offer preliminary evidence about validity and reliability of the modified tool for evaluating career choice among prospective healthcare professionals. The study supports the applicability of the HCC Scale.

## CONFLICT OF INTEREST

No conflict of interest has been declared by the authors.

## AUTHOR CONTRIBUTIONS

Each of the co‐authors has contributed significantly. AM: Study design, with contributions by AH. AM: Data collection. AH: Data analysis and interpretation. AM and AH: Manuscript preparation, revision of the article scientific content and drafting and the revision of the article. All authors gave final approval of the version to be published.

## RESEARCH ETHICS COMMITTEE APPROVAL

Research Ethics committee approval was granted for this study from the research committee at the university (No:40‐34‐0060). Participant identifiers or personal information were not collected as part of the study. Data were collected as anonymous individuals, and study data were transferred and stored in a secured place.

## Supporting information

Supplementary Material
