# Supplementary material for: Psychometric properties of factors influencing Healthcare Career Choice Scale
Source: Nurs Open. 2020 Jun 14;7(5):1588–96. doi: 10.1002/nop2.541 (PMC7424428; doi:10.1002/nop2.541)
Supplement: Supplementary file 1 — Supplementary Material [file NOP2-7-1588-s001.docx]

**Supplementary file I**

APPENDIX S1

|  | Original Item | Reworded Item | Inclusion/  Exclusion | Reasons for Inclusion/  Exclusion |
| --- | --- | --- | --- | --- |
| 1 | I am interested in teaching | I am interested in this health specialty | Included | Relevant/ essential |
| 2 | Part-time teaching could allow more family time | Part-time for this health specialty allows more family time | Excluded | Not essential  CVR < 0 |
| 3 | My friends think I should become a teacher | My friends encourage me to study this health specialty | Included | Relevant/ essential Loading values > 0.45 |
| 4 | As a teacher, I will have lengthy holidays | This health specialty allows me to have lengthy holidays | Excluded | Not essential  CVR < 0 |
| 5 | I have the qualities of a good teacher | I have the qualities needed to succeed in this health specialty | Included | Relevant/ essential  Loading values > 0.45 |
| 6 | Teaching allows me to provide a service to society | This health specialty allows me to provide a service to society | Included | Relevant/ essential  Loading values > 0.45 |
| 7 | I’ve always wanted to be a teacher | I’ve always wanted to select this health specialty | Included | Relevant/ essential  Loading values > 0.45 |
| 8 | Teaching may give me the chance to work abroad | This health specialty gives me the chance to work overseas | Excluded | Irrelevant:  I-CVI < 0.78 |
| 9 | Teaching will allow me to shape child/adolescent values | This health specialty allows me to shape community health | Excluded | Loading values <0.45 |
| 10 | I was unsure of what career I wanted | I was unsure of what health specialty I wanted | Included | Relevant/ essential  Loading values > 0.45 |
| 11 | I like teaching | I like this health specialty | Excluded | Loading values <0.45 |
| 12 | I want a job that involves working with children/adolescents | I want a job that involves working with patients of all ages | Included | Relevant/ essential  Loading values > 0.45 |
| 13 | Teaching will offer a steady career path | This health specialty will offer a steady career path | Included | Relevant/ essential  Loading values > 0.45 |
| 14 | Teaching hours will fit with the responsibilities of having a family | The working hours of this health specialty fits with the responsibilities of having a family | Excluded | Irrelevant:  I-CVI < 0.78 |
| 15 | I have had inspirational teachers | I have been amazed by some inspirational people at this health specialty | Included | Relevant/ essential  Loading values > 0.45 |
| 16 | As a teacher, I will have a short working day | This health specialty allows me to work part time | Excluded | Irrelevant:  I-CVI < 0.78 |
| 17 | I have good teaching skills | I have good skills specific for this health specialty | Included | Relevant/ essential  Loading values > 0.45 |
| 18 | Teachers make a worthwhile social contribution | This health specialty makes a worthwhile social health contribution | Included | Relevant/ essential  Loading values > 0.45 |
| 19 | A teaching qualification is recognised everywhere | This health specialty is recognised everywhere | Excluded | Loading values <0.45 |
| 20 | Teaching will allow me to influence the next generation | This health specialty will allow me to influence the next generation | Excluded | Not essential  CVR < 0 |
| 21 | My family think I should become a teacher | My family think I should study this health specialty | Included | Relevant/ essential  Loading values > 0.45 |
| 22 | I want to work in a child/adolescent-centred environment | I want to work in patient centred environment | Included | Relevant/ essential  Loading values > 0.45 |
| 23 | Teaching will provide a reliable income | This health specialty provides a reliable income | Included | Relevant/ essential  Loading values > 0.45 |
| 24 | School holidays will fit in with family commitments | This health specialty off days fit in with family commitments | Excluded | Not essential  CVR < 0 |
| 25 | I have had good teachers as role-models | In this health specialty, I have had seen some good role-models | Included | Relevant/ essential  Loading values > 0.45 |
| 26 | Teaching enables me to ‘give back’ to society | This health specialty enables me to ‘give back’ to society | Included | Relevant/ essential  Loading values > 0.45 |
| 27 | I was not accepted into my first-choice career | I was not accepted into my first-choice career | Included | Relevant/ essential  Loading values > 0.45 |
| 28 | 28. Teaching will allow me to raise the ambitions of underprivileged youth | This health specialty will allow me to serve sick, poor people, and the underprivileged. | Excluded | Loading values <0.45 |
| 29 | 29. I like working with children/adolescents | I like working with patient of all ages | Included | Relevant/ essential  Loading values > 0.45 |
| 30 | 30. Teaching will be a secure job | This health specialty will provide me a secure job | Included | Relevant/ essential  Loading values > 0.45 |
| 31 | 31. I have had positive learning experiences | I have had positive health care experience | Included | Relevant/ essential  Loading values > 0.45 |
| 32 | 32. People I’ve worked with think I should become a teacher | People I’ve met think I should study this health specialty | Included | Relevant/ essential  Loading values > 0.45 |
| 33 | 33. Teaching is a career suited to my abilities | This health specialty suited my abilities | Included | Relevant/ essential  Loading values > 0.45 |
| 34 | A teaching job will allow me to choose where I wish to live | This health specialty gives me the chance to select where I can to live | Excluded | Not essential  CVR < 0 |
| 25 | 35. I chose teaching as a last-resort career | I chose this health specialty as a last-resort career | Included | Relevant/ essential  Loading values > 0.45 |
| 36 | 36. Teaching will allow me to benefit the socially disadvantaged | This health specialty will allow me to benefit the socially needy people | Included | Relevant/ essential  Loading values > 0.45 |
| 37 | 37. Teaching will allow me to have an impact on children/adolescents | This health specialty will allow me to have an impact on people health | Included | Relevant/ essential  Loading values > 0.45 |
| 38 | Teaching will allow me to work against social disadvantage | This health specialty will allow me to work against social disadvantage | Excluded | Not essential  CVR < 0 |
| 39 | 39. Do you think teaching is well paid? | I think this health specialty is well paid | Included | Relevant/ essential  Loading values > 0.45 |
| 40 | 40. Do you think teachers have a heavy workload? | I think people working in this health specialty have heavy workload | Included | Relevant/ essential  Loading values > 0.45 |
| 41 | 41. Do you think teachers earn a good salary? | People working in this health specialty earns a good salary | Included | Relevant/ essential  Loading values > 0.45 |
| 42 | 42. Do you believe teachers are perceived as professionals? | I believe this health specialty is perceived as professionals | Included | Relevant/ essential  Loading values > 0.45 |
| 43 | Do you think teachers have high morale? | I think this health speciality have high morale? | Excluded | Loading values <0.45 |
| 44 | Do you think teaching is emotionally demanding? | I think this health specialty is emotionally demanding? | Excluded | Loading values <0.45 |
| 45 | Do you believe teaching is perceived as a high-status occupation? | I believe this health specialty is perceived as a high-status occupation | Included | Relevant/ essential  Loading values > 0.45 |
| 46 | Do you think teachers feel valued by society? | I think people working in this health specialty feel appreciated by society | Included | Relevant/ essential  Loading values > 0.45 |
| 47 | Do you think teaching requires high levels of expert knowledge? | I think this health specialty requires high levels of expert knowledge | Included | Relevant/ essential  Loading values > 0.45 |
| 48 | Do you think teaching is hard work? | I think this health specialty is hard | Included | Relevant/ essential  Loading values > 0.45 |
| 49 | Do you believe teaching is a well-respected career? | I think this health specialty is a well-respected career | Included | Relevant/ essential  Loading values > 0.45 |
| 50 | Do you think teachers feel their occupation has high social status? | I think people working in this health specialty feel their occupation has high social rank | Included | Relevant/ essential  Loading values > 0.45 |
| 51 | Do you think teachers need high levels of technical knowledge? | I think this health specialty need high levels of procedural knowledge | Included | Relevant/ essential  Loading values > 0.45 |
| 52 | Do you think teachers need highly specialised knowledge? | I think this health specialty requires high level of specialised knowledge | Included | Relevant/ essential  Loading values > 0.45 |
| 53 | How carefully have you thought about becoming a teacher? | I carefully thought before selecting my current health specialty | Included | Relevant/ essential  Loading values > 0.45 |
| 54 | Were you encouraged to pursue careers other than teaching? | I was encouraged to select other than this health specialty | Included | Relevant/ essential  Loading values > 0.45 |
| 55 | How satisfied are you with your choice of becoming a teacher? | I’m satisfied with my selection of my current health specialty | Included | Relevant/ essential  Loading values > 0.45 |
| 56 | Did others tell you teaching was not a good career choice? | I was told by others that selecting this health specialty was not a good decision | Included | Relevant/ essential  Loading values > 0.45 |
| 57 | How happy are you with your decision to become a teacher? | I’m happy with my decision about selecting this current health specialty | Included | Relevant/ essential  Loading values > 0.45 |
| 58 | Did others influence you to consider careers other than teaching? | I was influenced to consider other career than this health specialty | Included | Relevant/ essential  Loading values > 0.45 |
